# The neurobiological link between OCD and ADHD

**DOI:** 10.1007/s12402-014-0146-x

**Published:** 2014-07-14

**Authors:** Silvia Brem, Edna Grünblatt, Renate Drechsler, Peter Riederer, Susanne Walitza

**Affiliations:** 1 University Clinics of Child and Adolescent Psychiatry, University of Zürich, Neumünsterallee 9, 8032 Zurich, Switzerland; 2 Neuroscience Center Zurich, University of Zurich and ETH Zurich, Zurich, Switzerland; 3 Zurich Center for Integrative Human Physiology, University of Zurich, Zurich, Switzerland; 4 Department of Psychiatry, Psychosomatic and Psychotherapy, University Hospital of Würzburg, Füchsleinstr. 15, 97080 Würzburg, Germany

**Keywords:** OCD, ADHD, Neuroimaging, Genetics, Neuropsychology, Neurobiology, EEG, MRI, fMRI

## Abstract

Obsessive compulsive disorder (OCD) and attention deficit hyperactivity disorder (ADHD) are two of the most common neuropsychiatric diseases in paediatric populations. The high comorbidity of ADHD and OCD with each other, especially of ADHD in paediatric OCD, is well described. OCD and ADHD often follow a chronic course with persistent rates of at least 40–50 %. Family studies showed high heritability in ADHD and OCD, and some genetic findings showed similar variants for both disorders of the same pathogenetic mechanisms, whereas other genetic findings may differentiate between ADHD and OCD. Neuropsychological and neuroimaging studies suggest that partly similar executive functions are affected in both disorders. The deficits in the corresponding brain networks may be responsible for the perseverative, compulsive symptoms in OCD but also for the disinhibited and impulsive symptoms characterizing ADHD. This article reviews the current literature of neuroimaging, neurochemical circuitry, neuropsychological and genetic findings considering similarities as well as differences between OCD and ADHD.

## Introduction

Obsessive compulsive disorders (OCD) are typically characterized by the presence of recurrent, intrusive, and disturbing thoughts (obsessions) which often elicit anxiety or emotional stress followed by repetitive stereotypic behaviour or thoughts (compulsions) in order to neutralize the negative affects (American Psychiatric Association 1994). According to ICD-10 diagnostic classification, OCD consists of recurrent and persistent thoughts, behavioural patterns, ideas, and impulses that impose themselves against internal resistance, experienced by the patient as excessive and distressing. According to ICD-10 (World Health Organization 1996), OCD can be divided into “predominantly obsessional thoughts”, “predominantly compulsive acts”, or in a subtype of combination of both “obsessions and compulsions”. Fear of contamination, sexual, hypochondriac, and excessive thoughts including scruples/guilt are the most commonly reported obsessions and washing, repeating, checking, and ordering are the most commonly reported compulsions (Geller et al. 2001). In the DSM-5 (American Psychiatric Association 2013), OCD is newly classified into the diagnostic categories: “obsessive compulsive and related disorders” including “obsessive compulsive disorder”, and as related disorders e.g.“body dysmorphic disorder”, “hoarding disorder”, “hair pulling disorder” (trichotillomania). In the previous DSM-IV, OCD was classified as a subcategory of anxiety disorders. Therefore, in DSM-5, the specificity of the symptomatology including obsessions and compulsions with or without concomitant anxiety comes more to the centre of attention.

In DSM-IV, the diagnostic criteria for children with OCD differs from adults with OCD in regard to not having always insight into the senselessness of the obsessive behaviour. For adults, “showing insight into the senselessness of the symptoms” was one of the key criteria to differentiate OCD from psychosis; see also ICD-10. In DSM-5, clinicians even have to specify the degree of insight into the symptomatology: good/fair, poor, or absent insight. The presence of insight is of clinical importance because insight not only correlates with age but also with severity and positive therapy response (Walitza 2014). Clinicians also have to specify whether the patient has a current or past history of a tic disorder, this will be classified as a tic-related obsessive compulsive disorder (American Psychiatric Association 2013; Thomsen 2013; Walitza 2014). In the overview presented here, nearly all studies have used ICD-10 or DSM-IV for the classification of OCD in children and adults. Therefore, we want to anticipate that questions of confounding variables of the degree of insight and of the influence of previous or present tic disorders could not be answered by most of the previous findings and should be addressed using the DSM-5 criteria in future studies.

From an epidemiological point of view, OCD is the world’s fourth most common psychiatric disorder with a lifetime prevalence of 2–3 % (Flament et al. 1988; Robins et al. 1984; Zohar 1999). Delorme et al. (2005) considered the disorder to have a bimodal age distribution, with a first peak at age 11 and a second in early adulthood. Up to 50 % of all OCD cases emerge during childhood or adolescence (Flament et al. 1988; Nestadt et al. 2000). The course and outcome show high persistence rates with at least 40 % in retrospective and prospective follow-up studies in OCD (Stewart et al. 2004; Zellmann et al. 2009) whereby patients in remission with OCD often developed other psychiatric disorders and other psychiatric symptoms, exacerbated by the decrease of OCD symptoms (Stewart et al. 2004).

Attention deficit hyperactivity disorder (ADHD) is characterized by a persistent pattern of inattention and/or hyperactivity and impulsivity (American Psychiatric Association 1994). The symptomatology of ADHD interferes with functioning or development and has persisted (for at least 6 months) to a degree that is inconsistent with expected developmental level and that negatively impacts directly on social and academic/occupational activities. Manifestations of the disorder must be present in more than one setting (e.g. school, home, with friends or relatives). The symptoms vary depending on context, the higher the structure of the context (e.g. school), the higher seems to be the deviation from the average of normal behaviours. But it has to be taken into account that some forms of structure of school and homework can also help the child to focus more on important topics. According to DSM-5 criteria, ADHD is now classified as a neurodevelopmental disorder, which is a group of conditions with onset in the developmental period. These disorders (also including autism spectrum disorders and learning disorders) typically manifest in early development, often before the child enters grade school, and are characterized by developmental deficits that produce impairments of a broad range of social functioning (American Psychiatric Association 2013). Several inattentive or hyperactive–impulsive symptoms must have been present prior to age of 12 years (previously in DSM-IV, the symptomatology has to be present before age of 7 years). Therefore, we can assume that ADHD has on average an earlier onset of symptoms in comparison with OCD. DSM-5 distinguishes three different presentations: a predominantly inattentive presentation, a predominantly hyperactive/impulsive, and a combined presentation, if criteria for both inattention and hyperactivity–impulsivity are met. Although the lists of 18 symptoms from the DSM-5 (American Psychiatric Association 2013) and the ICD-10 (World Health Organization 1996) for ADHD are similar, ICD-10 is more specific as some symptoms must be present in all of the three dimensions (inattention, hyperactivity, and impulsivity). And hyperkinetic disorder (the nomenclature used in the ICD-10 that corresponds to ADHD in the DSM-5) is excluded if depression and/or anxiety disorders are also identified. In DSM-5, autism spectrum disorders, too, are no exclusion criteria for ADHD anymore.

ADHD has a higher prevalence than OCD and is overall one of the most common psychiatric disorders, with a worldwide prevalence of 5.2 % among children and adolescents (Polanczyk et al. 2007) using DSM-IV and DSM-5 criteria. ADHD persists into adulthood in 60–70 % of cases either as a residual or as a full clinical disorder (Biederman et al. 2000; Kessler et al. 2005). The estimated prevalence of adult ADHD in USA, Europe, and the Middle East is 3.4 % (range 1.3–7.3 %) (Fayyad et al. 2007; Kessler et al. 2006).

A study concerning the relationship between OCD and ADHD in children and adolescents using familial risk analysis provided further evidence of a familial relationship, in addition to unique aetiological factors for both OCD and ADHD (Geller et al. 2007a, b). This is of special importance because of the high comorbidity for both OCD and ADHD disorders. Sheppard et al. (2010) ascertained in a recent study an ADHD prevalence of 11.8 % in OCD-affected individuals. Masi et al. reported 2006 and 2010 in two samples of consecutive referred paediatric OCD patients a prevalence for comorbid ADHD of 17.1 % and 25.5 % respectively (Masi et al. 2006, 2010). The estimated rate of comorbid OCD among children with ADHD is 8 % (Geller et al. 2000). In our studies, ADHD was the most common comorbidity in early-onset OCD, in which tic and Tourette syndrome were exclusion criteria (Walitza et al. 2008). In this study, comorbidity of ADHD in early-onset OCD seems also to predict a higher severity of OCD and a higher grade of persistence of OCD in a prospective follow-up period (Walitza et al. 2008).

This article aims to summarize and compare findings of structural and functional abnormalities, neuropsychological aspects, biochemical and genetic studies on OCD and ADHD. Despite the high comorbidity of both disorders, only very few studies have investigated both together or have directly compared both within the same study. This article shows in the following those rare studies investigating both disorders and also results of each disorder alone (focusing on meta-analyses, if possible) with a following contrast made by the authors of the present review. Some of the results showed shared aetiological factors and mechanisms for both disorders, whereas other findings differentiated between ADHD and OCD. The aim of this overview is to help to understand the aetiology of psychiatric disorders, in particular concerning ADHD and OCD.

## Structural and functional abnormalities in OCD and ADHD

A major aim in recent years has been to shed light on the relationship between clinical symptoms of ADHD and OCD and the underlying brain structure, function, and connectivity. Extensive neuroimaging literature exists on the comparison of either disorders with matched healthy controls, and a variety of methods have been used to assess brain structure, metabolism, and the spatial and temporal organization of brain networks. The timing of information processing is commonly studied with brain imaging methods that exhibit a high temporal resolution in millisecond time range, such as electroencephalography (EEG) or alternatively by magnetoencephalography. Details about brain structure and the spatial activation pattern are best assessed by (functional) magnetic resonance imaging or positron emission tomography (PET). Here, we summarize the most consistent abnormalities found in brain structure and functional activation patterns revealed by structural magnetic resonance imaging, functional magnetic resonance imaging (fMRI), and event-related potentials (ERP) studies for both ADHD and OCD. We focused on similarities and differences of affected brain regions reported across these patient groups and specifically point to meta-analytic studies and their findings. Concluding this section, we summarize the results of the only study that directly compared functional neuroimaging data of paediatric patients with ADHD to patients with OCD (Rubia et al. 2010, 2011).

There is considerable evidence for structural differences in the brains of ADHD patients when compared to age-matched healthy controls. Most of these findings are relatively inconsistent and depend on age and/or medication with stimulants. Regarding age, it has been shown that patients with ADHD show a regional delay in the maturation of cortical thickness, especially in regions responsible for cognitive control such as attention, working memory, inhibition and evaluation of reward contingencies in the prefrontal cortex (Shaw et al. 2007), and a slightly earlier maturation of the primary motor cortex. The authors thus suggested that the abnormal development of cognitive control and motor areas may drive the poor control of motor activity (Shaw et al. 2007).

**Fig. 1:**
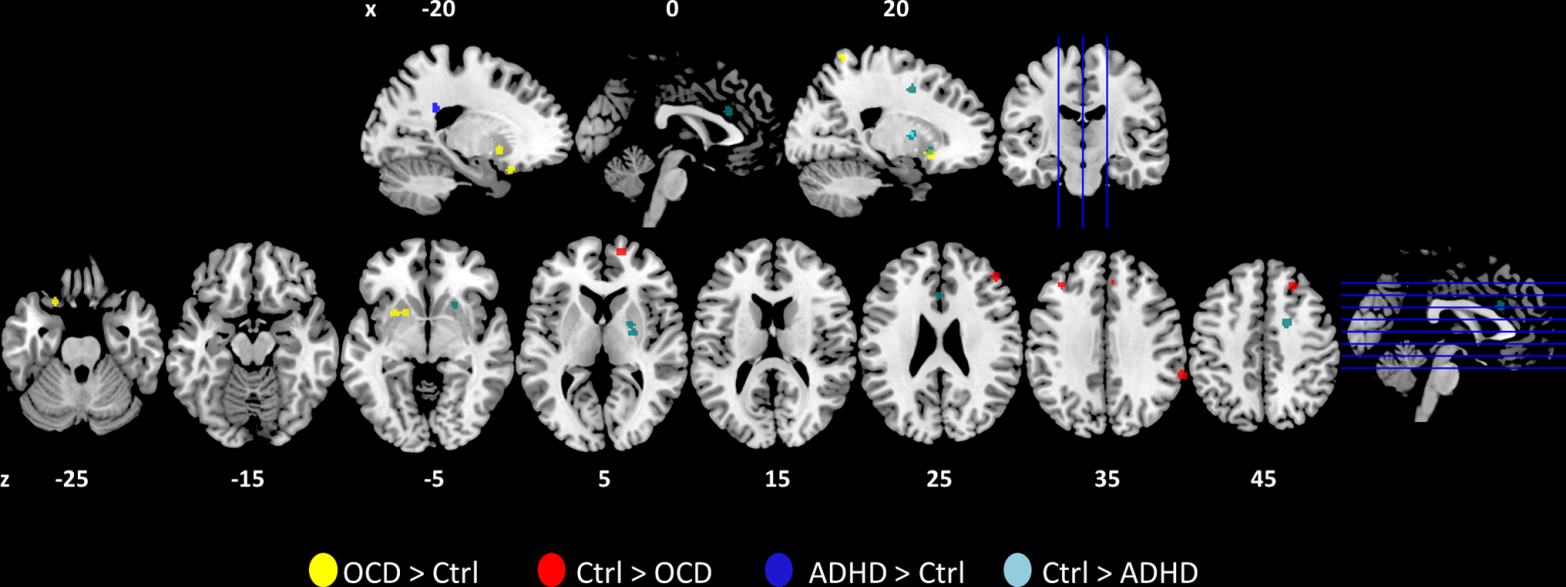
The centres of the clusters that showed differences in grey matter volumes of patients with ADHD or OCD in five recent meta-analyses (Ellison-Wright et al. 2008; Frodl and Skokauskas 2012; Nakao et al. 2011; Rotge et al. 2010; Radua and Mataix-Cols 2009) are plotted as spheres (*r* = 4 mm) on sagittal (*top*) and axial slices (*bottom*) using MRICron (Rorden et al. 2007). Alterations in the grey matter volumes of patients with ADHD are shown in *blue* and of patients with OCD in *red/yellow*. Nicely visible are the differences in the volume of the basal ganglia in ADHD (reduced volume: *light blue*) and OCD (increased volume: *yellow*) as compared to controls

Recent meta-analyses summarized the most consistently replicated structural differences between ADHD patients and controls. They emphasize the reduced volume of the basal ganglia, especially the lentiform nucleus (globus pallidus and putamen) (Ellison-Wright et al. 2008; Nakao et al. 2011) and the caudate (Ellison-Wright et al. 2008; Frodl and Skokauskas 2012; Nakao et al. 2011; Valera et al. 2007) seen in ADHD patients. More specifically, the abnormal size of the caudate seems to depend on age and has been reported to be most prominent in prepubescents with ADHD (Carrey et al. 2012; Castellanos et al. 2002; Mahone et al. 2011). Furthermore, partial normalization of the basal ganglia volume was found when ADHD patients were treated with stimulant medication (Nakao et al. 2011). Apart from an increased volume of the posterior cingulate cortex, other often reported abnormalities in the form of reduced cortical thickness and/or volume of the total brain (Castellanos et al. 1996, 2002; Hill et al. 2003), corpus callosum (Giedd et al. 1994; Hill et al. 2003), prefrontal (Hill et al. 2003; Shaw et al. 2007; Sowell et al. 2003), temporal (Sowell et al. 2003), and cerebellar cortex (Castellanos et al. 1996, 2002) did not reach significance in the most recent meta-analysis of Nakao et al. (2011). Similarly to ADHD patients, OCD patients showed consistent deviations in the volume of the basal ganglia (Piras et al. 2013, Rotge et al. 2010). Older studies were inconsistent on whether this structure showed enlarged (Baxter et al. 1987, 1988; Scarone et al. 1992), decreased (Luxenberg et al. 1988; Robinson et al. 1995), or normal volume (Aylward et al. 1996; Jenike et al. 1996; Szeszko et al. 2008) in patients (for comprehensive reviews see: Saxena and Rauch 2000; Friedlander and Desrocher 2006; Huey et al. 2008). A review (Piras et al. 2013) and two meta-analyses (Radua and Mataix-Cols 2009, Rotge et al. 2010) pointed to relatively consistent patterns of increased basal ganglia volume in more recent studies. Besides alterations in basal ganglia, the meta-analyses of structural differences between OCD patients and healthy controls also detected reduced grey matter in the frontal eye fields, the dorsolateral prefrontal cortex, and the medial frontal cortex, including the anterior cingulate cortex (ACC) (Radua and Mataix-Cols 2009), the left and right orbito-frontal cortex (OFC), and the supramarginal gyrus (Rotge et al. 2009, 2010). An overview of the regions with altered grey matter volumes in patients, as reported in the above-mentioned meta-analyses (Ellison-Wright et al. 2008; Frodl and Skokauskas 2012; Nakao et al. 2011; Rotge et al. 2010; Radua and Mataix-Cols 2009), is given in Fig. 1.

The structural abnormalities thus nicely converge with the neurobiological models, suggesting a failure of cortico-striato-thalamico-cortical (CST) circuit function in ADHD and OCD patients (van den Heuvel et al. 2010).

In line with structural findings, the results of a meta-analysis in adult OCD patients pointed to activation differences in corresponding functional brain networks (Brem et al. 2012; Menzies et al. 2008). Apart from clear support for abnormal activation in orbito-fronto-striatal regions, lateral frontal, anterior cingulate, middle occipital, and parietal cortices, the cerebellum also exhibited altered activation in cognitive tasks (Menzies et al. 2008). A preliminary meta-analysis in paediatric OCD patients largely converged with these findings, even though the direction of activation differences yielded partly opposing results (Brem et al. 2012). The alterations in the activation pattern of ADHD differed between children and adults as shown in a recent meta-analysis. Both adults and children yielded hypoactivation in fronto-parietal executive function networks that have been related to the well-known deficiencies in performing goal-directed executive processes and hyperactivation in the default network, suggesting a faulty interregulation between the networks activated during tasks and the default network (Cortese et al. 2012). Further alterations in activation have been detected in the ventral attention and the somatomotor networks in children and in the visual and dorsal attention systems in adults (Cortese et al. 2012).

The ACC converged in showing both structural and functional alterations in ADHD and OCD patients as compared to healthy controls. The ACC has an important role in attentional and emotional processes (Bush et al. 2000; van Veen and Carter 2002a) but, moreover, is also involved in conflict detection and evaluation (Botvinick et al. 1999; Carter et al. 2000; Durston et al. 2003a; Ridderinkhof et al. 2004; Ullsperger and von Cramon 2004) and performance monitoring (Albrecht et al. 2010, 2008; Maltby et al. 2005; Ridderinkhof et al. 2004; Ullsperger and von Cramon 2004, 2006; Ursu et al. 2003). The characteristic overactivation of this brain structure in OCD patients points to excessive activity in the action monitoring system when processing errors or correct responses in high-conflict trials (Maltby et al. 2005; Ursu et al. 2003) and has thus been suggested to reflect a neural correlate of the patients’ continuing sense that something is not quite “right” (review see: Aouizerate et al. 2004) and requires correction. In contrast, performance monitoring and error and conflict processing in ADHD patients seem to induce less activation in the ACC as demonstrated by fMRI (Durston et al. 2003b; Rubia et al. 2010, 2005; Tamm et al. 2004) and ERP studies (Albrecht et al. 2010, 2008; Groen et al. 2008; Liotti et al. 2005; McLoughlin et al. 2009; van Meel et al. 2007). The high time resolution of ERPs allows one to better disentangle conflict-related from actual error-related effects through response-locked averaging. An electrophysiological correlate of ACC activity which can be detected in the ERP is the response-locked error-related negativity (ERN, Ne) occurring at around 50–150 ms after error commission. Holroyd and Coles suggested that the Ne emerges when a phasic error signal originating from the mesolimbic dopamine system is processed in the ACC in order to modify performance and update behaviour (Holroyd and Coles 2002). The characteristic fronto-central negativity of this Ne in OCD and ADHD has shown to differ from controls. The more pronounced Ne amplitudes found in children (Endrass et al. 2008; Grundler et al. 2009; Johannes et al. 2001) as well as in adults with OCD (Hajcak et al. 2008; Santesso et al. 2006) are in line with the stronger ACC activity seen in fMRI studies. In contrast to OCD patients with their overactive response monitoring system, patients with ADHD show diminished Ne and ACC activity (Albrecht et al. 2010, 2008; Bush et al. 1999; Durston et al. 2003b; Groen et al. 2008; Liotti et al. 2005; McLoughlin et al. 2009; Rubia et al. 1999; Tamm et al. 2004; van Meel et al. 2007). A very recent meta-analysis of the Ne in adolescent and adult patients with ADHD clearly supported previous findings of Ne attenuation (Geburek et al. 2013) and provides further evidence for a deficit in cognitive control mechanisms.

Anterior cingulate cortex activity has not only been related to performance monitoring and conflict anticipation (Sohn et al. 2007) or processing but seems crucial for inhibition processes as well. Thus, ACC overactivity in OCD may, in addition to excessive performance monitoring, also indicate a failure in the inhibition of prepotent responses in OCD patients causing the repetitive behaviour of compulsions (Maltby et al. 2005). The neurobiological correlates of inhibitory deficits in ADHD contrast to the ones of OCD patients because deficient inhibition mainly emerged as ACC hypoactivation in STOP or flanker tasks (Konrad et al. 2006; Pliszka et al. 2006; Rubia et al. 1999). Hypoactivation and corresponding deficient inhibition in ADHD seem responsible for inappropriate higher-order motor control mechanisms (Rubia et al. 1999). On the other hand, and probably depending on the task, hyperactivation can occur in similar cortical and subcortical structures of ADHD patients (Durston et al. 2003b; Schulz et al. 2004).

ERPs associated with inhibition processes include the fronto-central N2 (or N200) negativity after 200–300 ms and a later (350–600 ms) fronto-central P3 (or P300) positivity. These ERPs have usually been studied with STOP and Go/Nogo tasks (Brandeis et al. 1998; Falkenstein et al. 1999; Kopp et al. 1996). More pronounced amplitudes characterize the N2 and the P3 in response to trials requiring behavioural inhibition, such as Nogo as compared to Go trials. Regarding the N2, the findings in OCD patients are inconsistent: depending on the task, authors reported comparable (Di Russo et al. 2000), enhanced (Ruchsow et al. 2007), or reduced amplitudes and different topographies (Kim et al. 2007) to Nogo trials in the N2. For ADHD, the deficits in the right frontal N2 seemed to dominate in STOP tasks with a high inhibition demand (Albrecht et al. 2005; Dimoska et al. 2003; Liotti et al. 2005; Pliszka et al. 2000). The N2, generated in the caudal region of the ACC, precedes the actual motor response during conflicting trials (van Veen and Carter 2002b). Even though the N2 has traditionally been associated with inhibitory processes, the modulation of its amplitude by conflict level indicated that the N2 primarily reflects conflict processing rather than motor inhibition (Donkers and van Boxtel 2004; Enriquez-Geppert et al. 2010; van Veen and Carter 2002a).

Accordingly, a recent study showed that the conflict-induced amplitude increase in the N2 was significantly reduced in children with ADHD. Furthermore, non-affected siblings exhibited intermediate amplitudes in between ADHD subjects and healthy peers (Albrecht et al. 2010, 2008). The subsequent P3 has been associated with phasic inhibitory motor control mechanisms emerging from the right frontal cortex (Strik et al. 1998). The Nogo P3 typically shows an anteriorisation (Nogo anteriorisation) of its central positivity in contrast to Go trials (Fallgatter and Strik 1999) which has been related to ACC activity and prefrontal response control (Fallgatter et al. 2005). Most often, the P3 in visual Go/Nogo tasks did not differ in amplitude between patients with OCD and controls, even though its latency (Johannes et al. 2001) and topography sometimes differed (Herrmann et al. 2003; Malloy et al. 1989). In contrast, studies on ADHD show quite consistent differences in the Nogo P3. Both children and adults with ADHD showed reduced amplitudes and diminished Nogo anteriorisation in the narrow time window preceding actual response inhibition in the P3 (Fallgatter et al. 2004, 2005).

A direct comparison of functional activation between ADHD and OCD patients is only available from a paediatric sample and for tasks concentrating on executive functions. The group of Rubia examined interference inhibition, selective attention (Rubia et al. 2011), motor response inhibition, and cognitive flexibility (Rubia et al. 2010) using event-related fMRI. Common dysfunction in paediatric patients with ADHD or OCD as compared to controls emerged as hypoactivation in mesial frontal areas: reduced activity in patients was found in the right orbito-frontal cortex and ACC for successful inhibition, in the left medial frontal cortex and ACC for failed inhibition, and finally in bilateral inferior frontal and insular cortices extending also to the left premotor cortex, right superior temporal areas, and putamen for cognitive switching processes (Rubia et al. 2010). Further interference inhibition and selective attention in a modified Simon task was associated with reduced activity in supplementary motor areas, the ACC and superior parietal cortices in both patient groups (Rubia et al. 2011).

Disorder-specific hypoactivation was predominantly found for ADHD patients and again was condition and task specific: activation in the left putamen, caudate, cingulate, and parietal cortex was reduced as compared to healthy controls and OCD patients during cognitive switching (Rubia et al. 2010) and in the Simon task (Rubia et al. 2011). The pattern of functional deficits in the basal ganglia thus corresponds to the consistent structural abnormalities reported for ADHD (Ellison-Wright et al. 2008; Nakao et al. 2011). Failed stop trials were furthermore associated with diminished right middle and inferior prefrontal activation in ADHD as compared to healthy controls and OCD patients (Rubia et al. 2010).

Disorder-specific alterations in brain activation of children with OCD were less pronounced. They differed from healthy controls and ADHD patients only in the oddball condition by showing reduced activation in the right superior and middle frontal gyri of the dorsolateral prefrontal cortex (Rubia et al. 2011). It however remains questionable whether this finding is confounded by effects of medication and symptom severity in the relatively small group of partly remitted OCD patients with only low symptom levels (Rubia et al. 2010).

In summary, deficits in the cortico-striato-thalamic circuits responsible for cognitive control and performance monitoring processes are characterized in both neuropsychiatric conditions: ADHD and OCD. According to the nature of their symptoms situated at the opposite ends of the impulsive–compulsive spectrum, they either exhibit hypo- or hyperactivation of affected brain structures such as basal ganglia or the mesial frontal cortex (Rubia et al. 2010; Carlsson 2000). Affected CST networks and the resulting deficits in cortical inhibition and/or disinhibition may thus, on the one hand, facilitate the perseverative, compulsive behaviours seen in OCD patients but, on the other hand, also explain the disinhibited, impulsive, inattentive behaviour of ADHD patients.

## Neuropsychological aspects in OCD and ADHD

Neuropsychological deficits have been described for adults with OCD in the domain of executive functions, especially impaired inhibition, impaired control of interference/conflict, diminished cognitive flexibility in switching, and cognitive alternation tasks (Abbruzzese et al. 1997; Aycicegi et al. 2003; Chamberlain et al. 2006, 2007; Gu et al. 2008; Remijnse et al. 2006; Veale et al. 1996; Watkins et al. 2005), as well as impaired planning (Cavedini et al. 2001; Chamberlain et al. 2007; Mataix-Cols et al. 2002; Nielen and Den Boer 2003; van den Heuvel et al. 2005). Several studies report problems in visuo-spatial working memory (Moritz et al. 2011; Savage et al. 1996), implicit learning tasks (Goldman et al. 2008; Kathmann et al. 2005), and, less consistently, visuo-spatial learning (see Simpson et al. 2006; Penades et al. 2005; Savage et al. 1999; meta-analyses by Olley et al. 2007; Abramovitch et al. 2013; but see analysis by Harkin and Kessler 2011). Deficits in motor and processing speed have also been reported (Burdick et al. 2008) with slower responding in OCD compared to controls. Only few studies have investigated neuropsychological performances in paediatric OCD, which partly replicate findings from OCD in adults on impaired inhibition (Rosenberg et al. 1997; Woolley et al. 2008; but see Beers et al. 1999; Shin et al. 2008; Ornstein et al. 2010), cognitive flexibility (Shin et al. 2008; Ornstein et al. 2010; but see Beers et al. 1999), planning (Andres et al. 2007; Behar et al. 1984; Cox et al. 1989; but see Beers et al. 1999), and visual memory (Andres et al. 2007; but see Behar et al. 1984 ; Cox et al. 1989). Poor fine motor skills and visuo-spatial skills in paediatric OCD seem to predict the persistence of OCD into adulthood (Bloch et al. 2011). Neuropsychological deficits have also been found in remitted OCD (Chamberlain et al. 2007; Rao et al. 2008) or in close relatives (Chamberlain et al. 2008), which suggests that they are trait markers or endophenotypes of OCD. In ADHD research, a large number of studies have investigated neuropsychological functioning both in children and adults. There is agreement that neuropsychological deficits in ADHD are heterogeneous and that only about 30–50 % of ADHD patients present clinically relevant neuropsychological impairment when assessed by objective tests (Biederman et al. 2004; Lambek et al. 2011; Loo et al. 2007; Nigg et al. 2005). Meta-analyses report consistent deficits in the domains of response inhibition, vigilance, planning, and working memory (Huang-Pollock et al. 2012; Kasper et al. 2012; Willcutt et al. 2005) in children; verbal fluency, inhibition, set-shifting (Boonstra et al. 2005), and focused and sustained attention (Balint et al. 2009) in adults; and interference control and enhanced reaction time variability in both (Kofler et al. 2013; Lansbergen et al. 2007).

### Cognitive style and error processing

Metacognition is impaired in OCD and the cognitive style of patients with OCD is marked by doubts about their own performance (Hermans et al. 2008; but see Moritz et al. 2011) and an overly cognitive self-consciousness which might interfere with effortful encoding (Exner et al. 2009; Kikul et al. 2011). The opposite phenomenon has been observed in a subgroup of children with ADHD (Hoza et al. 2002; Owens et al. 2007; Rizzo et al. 2010) and, to a lesser extent, in adults (Jiang and Johnston 2012; Knouse et al. 2005): they show a characteristic overestimation of competence and/or performance, which is known as “positive illusory bias”. Children with ADHD show impaired error monitoring and a lack of characteristic post-error slowing in inhibitory response tasks (Schachar et al. 2004). As mentioned above, in electrophysiological studies, this impairment is reflected by a diminished amplitude of the error-related negativity (Albrecht et al. 2008). In OCD patients, in contrast, error-related negativity is enhanced (Endrass et al. 2008).

### Decision-making and reward-related processing

During the last years, neuropsychological OCD research has focussed on decision-making and reward-related learning, with inconsistent results for tasks with implicit or complex reward contingencies (Cavedini et al. 2002; Chamberlain et al. 2007; Dittrich and Johansen 2013; Lawrence et al. 2006; Nielen et al. 2002; Watkins et al. 2005). However, a recent study based on large groups indicates that impaired decision-making might be a key feature in OCD (da Rocha et al. 2011) and already present in childhood (Kodaira et al. 2012). More consistent deficits have been found in reversal learning and response cost paradigms: patients with OCD persist in their response strategy in spite of changed reward contingencies (Chamberlain et al. 2008; Remijnse et al. 2006), or with the risk of smaller gains (Chamberlain et al. 2007). It has been hypothesized that impaired learning from feedback (Nielen et al. 2009) might be at the origin of impaired cognitive flexibility or an overcautious cognitive style. However, learning from feedback seems intact when it leads to the avoidance of negative consequences (Endrass et al. 2011). Similarly, ADHD research on decision-making and learning from feedback has yielded mixed results, but there is agreement that sensitivity to reinforcement is altered (Luman et al. 2010; Modesto-Lowe et al. 2013). Children with ADHD may opt for a smaller reward when this helps them to avoid waiting time (“Delay Aversion” (DA)) (Bitsakou et al. 2009; Sonuga-Barke 2002), and they are more responsive to immediate than to delayed rewards (Sagvolden et al. 2005).

### Contrasting OCD and ADHD

Vloet et al. (2010) directly compared neuropsychological performances in adolescents with OCD or ADHD using a serial reaction time task, developed to assess implicit sequence learning, and a DA task in order to assess abnormal motivational processes. Subjects with ADHD chose less frequently the larger, more delayed reward compared to those with OCD and controls. Subjects with OCD showed impaired implicit learning. In contrast, the ADHD group was unimpaired in their implicit learning behaviour and the OCD group was not characterized by a DA style. Within the OCD group, severity of obsessions was associated with implicit learning deficits and impulsive symptoms with DA in the ADHD group. In other tasks and domains, however, the differentiation of the two disorders is less evident: In a meta-analysis of the Stop-Task, Lipszyc and Schachar (2010) compared studies with different psychiatric populations and found medium effect sizes for deficits in stop signal reaction time for both ADHD (*g* = 0.62) and OCD (*g* = 0.77), reflecting the diminished speed of the inhibitory process. In a recent study comparing adults with ADHD and OCD, Abramovitch et al. (2012) found similar deficits of response inhibition in both disorders, but higher self-reported impulsivity in the ADHD group. These authors view executive function deficits in OCD as an epiphenomenon caused by the overflow of intrusive thoughts. According to their executive overload model, cognitive deficits in OCD patients result from the attempt to gain control over automatic processes in order to reduce impulsive behaviour and lapses of attention. This leads to increased consummation of cognitive resources and in return to diminished effective control.

### Neuropsychological profiles of subgroups

In ADHD as in OCD, apparent inconsistencies in neuropsychological findings have been explained by the existence of disorder-specific subgroups with differing neuropsychological key deficits. In OCD, the paradox of concurrent findings of diminished inhibitory control and slow responding has been related to different symptom dimensions, e.g. contrasting obsessional plus “slow decision-making” symptoms vs. compulsive plus “inhibition deficit/rigid” symptoms (da Rocha et al. 2008; Friedlander and Desrocher 2006; Mataix-Cols et al. 2005). Differential neuropsychological profiles have been described e.g. in patients with obsessions related to checking, symmetry/ordering, or contamination (Hashimoto et al. 2011; Jang et al. 2010), in “checkers” compared to “washers” (Nedeljkovic et al. 2009) and in different types of compulsion (Fineberg et al. 2010). Similarly, contradicting neuropsychological findings in ADHD have been explained by different neurobiological origins and the ensuing heterogeneity of neuropsychological symptoms. Accordingly, neurocognitive problems in children with ADHD may be due to dysfunctional executive functions, to a dysfunctional motivational/reward system, or to a combination of both (Sonuga-Barke 2005). Recently, impaired time processing has been suggested as a possible third pathway (Sonuga-Barke et al. 2010).

### Comorbidity and neuropsychological deficits

Comorbidity may attenuate or enhance neuropsychological symptoms. Compared to “pure” ADHD, comorbid ADHD with OCD seems to go along with attenuated neuropsychological impairment (Arnold et al. 2005). The impact of comorbid ADHD on neuropsychological deficits in OCD patients, compared to “pure” OCD, has not yet been systematically investigated. However, comorbid ADHD in childhood-onset OCD seems often to be associated with hoarding symptoms (Fullana et al. 2013; Sheppard et al. 2010), and the neuropsychological profile of patients with hoarding closely resembles that of ADHD inattentive subtype patients, with symptoms of diminished sustained attention (Tolin et al. 2011). Neuropsychological deficits have been found to be less pronounced in OCD with comorbid tics or Tourette disorder (Chang et al. 2007; Rankins et al. 2005; Watkins et al. 2005), or differentially affected in the presence of disorders from the autistic spectrum (Zandt et al. 2007, 2009). In childhood ADHD, comorbidity with tics/Tourette seems to result in a combination of neuropsychological problems associated with both disorders (Greimel et al. 2011; Shin et al. 2001). When ADHD symptoms are present in high-functioning autism or Asperger syndrome, neuropsychological deficits may correspond to a combination of characteristics from both disorders, with more severe deficits than in autism alone (Yerys et al. 2009), but attenuated compared to “pure” ADHD (Sinzig et al. 2008; but see van der Meer et al. 2012).

## Functional neurochemistry of neurotransmitter circuitry systems in OCD and ADHD

Neurotransmitter interactions and homeostasis are essential features of normal behaviour. The seminal work of Albin et al. (1989), Alexander and Crutcher (1990), DeLong et al. (1985) and others implicated various “loop-systems” for the various phenotypes of human and animal behaviour. The CST circuitry and its subregional connections play a major role; hyperkinetic disturbances are based on reduced stimulation of the substantia nigra pars reticulata and the medial globus pallidus by the subthalamic nucleus (NST). This can be caused by a disturbance of the NST (ballism) or via reduced striatal inhibition of the lateral globus pallidus (choreatic movement) (Reiner et al. 1988). In both cases, the result is disinhibition of the thalamus which in consequence leads to reduced feedback on cortical areas (Crossman et al. 1985). Sensory impulses may be causal for the hyperkinetic movements. Healthy individuals are able to suppress such reactions (Albin et al. 1989). Striatal nerve cells, which are sensitive for such sensory stimuli (Crutcher and DeLong 1984), seem to play a key role in uncontrolled sensory inputs (Paloyelis et al. 2012).

In the past, electrophysiological studies formed the basis in the development of circuitry systems. More recent experimental work has aimed at elucidating the interaction of neurotransmitters in such “loops”. Such work has been reviewed in detail by Berger and Riederer (1992), Mehler-Wex et al. (2006), Rommelfanger and Wichmann (2010), Carlson et al. (2006), and many others. They all focus primarily on disturbances of circuitry systems in motor behaviour (hypo- and hyperkinetic syndromes such as Parkinson’s disease (PD), Huntington’s Chorea, ADHD) and less often on mood disorders, such as schizophrenia.

Due to seminal studies by the Viennese Oleh Hornykiewicz and Walther Birkmayer in the early 1960s, which found that a deficiency of dopamine in the striatum of patients with PD could be substituted by L-DOPA, thereby improving major symptoms of PD, akinesia, rigidity, and tremor substantially, most work in the past has concentrated on “dopamine” as a major neurotransmitter for “motoricity” and “reward”. There is a structural abnormality in children with ADHD supporting the hypothesis of a nigro-striatal defect (Romanos et al. 2010) underlying motor behavioural alterations in this disorder. Additionally, impulsivity, attention deficit, and mood changes seem to be related to modulating circuits involving other brain areas.

Not only dopaminergic drugs can influence impulsivity and compulsive behaviour. This is shown by recent experiments demonstrating that blockade of noradrenergic α2 receptors improves sustained attention and response inhibition while α1- and β1- and β2-adrenergic receptor blockade disrupted go performance and sustained attention (Bari and Robbins 2013). Such data clearly demonstrates noradrenergic neurotransmitter interactions within mood circuitries. This is substantiated by genetic models using dopamine transporter (*DAT*), norepinephrine transporter (*NET*), and serotonin transporter (*SERT*) knockout mice. These observations by Gallagher et al. (2013) correlate with behavioural studies indicating that *SERT* knockout mice display anxiety-like phenotypes, while *NET* knockouts and to a lesser extent *DAT* knockout mice display antidepressant-like phenotypic features (Gallagher et al. 2013).

The D4 receptor is enriched in the prefrontal cortex and thus has been implemented in mood disorders. Yuen et al. (2013) described the restoration of glutamatergic transmission by D4 receptors in stressed animals. It is of interest that attention deficits can be induced by blocking N-methyl D-aspartate (NMDA) receptors in the prefrontal cortex and this is associated with enhanced glutamate release and cyclic adenosine monophosphate response element binding phosphorylation (Pozzi et al. 2011). Increased concentrations of glutamate in the ACC of subjects with borderline personality disorder with and without comorbid ADHD have been detected in cross-sectional proton magnetic resonance spectroscopy studies (Hoerst et al. 2010; Rusch et al. 2010). The close relationship and interaction between dopaminergic and glutamatergic neurotransmission has been described in the late 1980s. From our own work, we concluded that limbic dopaminergic activity is associated with psychotic states, while the same behaviour can be seen with reduced NMDA receptor channel blockade (Berger and Riederer 1992). Quality, quantity, regional, and subregional occurrence of such interaction determines the phenotype of symptomology.

Another neurotransmitter of interest is serotonin. This neurotransmitter is a “modulator”, like a “fine tuning” system, that interacts with many other neurotransmitters such as dopamine and glutamate. Therefore, it is not unlikely to assume that serotonin is also involved e.g. in impulsivity (Dalley and Roiser 2012). As summarized by Hunt et al. (1982) the aminergic neurotransmitter hypotheses for ADHD have been developed in particular in the 1980s. These studies included the measurement of serotonin in blood (Haslam and Dalby 1983) as well as measurements of total, free, and bound tryptophan (Ferguson et al. 1981; Hoshino et al. 1985; Irwin et al. 1981) with in part discrepant results. More recent studies demonstrate an inverse relationship between trait impulsivity and the acute tryptophan depletion effect on reactive aggression after low provocation in patients with adolescent and adult ADHD (Kotting et al. 2013; Zimmermann et al. 2012). While there seems to be a relationship between acute tryptophan depletion and attentional performance in adult patients with ADHD (Mette et al. 2013), there is no such effect on verbal declarative memory in young patients with ADHD (Zepf et al. 2013) and on processing affective prosody in male adults with ADHD (Grabemann et al. 2013).

As already elaborated in chapter 2, the fronto-striatal loop plays a major although not exclusive role in OCD symptomology. This correlates with findings of e.g. Gonçalves et al. (2011) and the fact that frontal-subcortical circuits are involved in behavioural aspects (Cummings 1995).

Furthermore, an OFC pathological neural substrate underlying olfactory identification impairment, impulsivity, and OCD has been described by Bersani et al. (2013). In contrast, deficits in visual memory, executive functions, and attention indicate that regions outside of the OFC may be involved in OCD (Bersani et al. 2013). The specific involvement of the NST in emotional processes in humans has been further described by Buot et al. (2012). These authors showed that the ventral part of the NST processes the emotional violence of stimuli independently of the motor context and that dopamine enhances the processing of pleasant information (Buot et al. 2012). In addition, experimental work in monkeys suggest that overactivity of the ventral anterior and medial dorsal nuclei of the thalamus provokes compulsive-like behaviours and neurovegetative manifestations including anxiety in patients with OCD (Rotge et al. 2012).

 While plasma catecholamines and metabolites are not changed in OCD (Benkelfat et al. 1991) concurring with no change in cerebrospinal fluid (CSF) concentrations of biogenic amines and metabolites (Leckman et al. 1995), supersensitive beta-adrenergic receptors are measured by detecting adenylate cyclase activity in platelets of OCD patients (Marazziti et al. 2009).

The role of dopamine in OCD is becoming more significant especially since the augmentation of dopaminergic receptor antagonists shows positive treatment responses (Koo et al. 2010). In fact, PET-studies labelling D1 receptors have demonstrated down regulation in the striatum (Olver et al. 2009) and ACC (Olver et al. 2010). Imaging studies also show a reduction of D2 binding (Nikolaus et al. 2010). Increased dopaminergic activity might contribute to these findings. This corresponds to the findings that metabolism via catecholamine-O-methyltransferase (*COMT*) and monoamine-oxidase-A (*MAO*-A) demonstrates polymorphisms in both these genes in males as shown by a meta-analysis by Taylor (2013), while *DAT1*- and *DRD3*-polymorphisms could not be identified so far.

As with disturbances in the glutamatergic system, there is evidence for significant increases of both glutamate and glycine in the CSF of OCD patients (Bhattacharyya et al. 2009). As indicated by a multivariate analysis of variance, CSF glycine concentrations were even higher in those OCD patients who had autoantibodies compared with those without (Bhattacharyya et al. 2009).

Using proton magnetic resonance spectroscopy (MRS), female OCD patients had a significantly reduced concentration of glutamate–glutamine in subareas of the ACC. In addition, male and female OCD patients had higher concentrations of myoinositol-containing compounds in their right rostral and dorsal ACC (Yucel et al. 2008). However, in the medial prefrontal cortex (MPFC), voxel-based imaging including the pregenual ACC (Simpson et al. 2012) could not detect glutamate–glutamine abnormalities in unmedicated OCD adults, while MPFC gamma-aminobutyric acid (GABA) levels were decreased. MRS studies measuring N-acetylaspartate showed significantly lower concentrations in the left head of the caudate nucleus (HOC) in non-medicated patients with OCD compared to medicated ones, while preliminary data suggest a correlation of behaviour therapy with a decrease in glutamate–glutamine in the right HOC (Whiteside et al. 2012).

It remains to be clarified in further studies whether OCD is a hyperglutamatergic and ADHD a hypoglutamatergic condition with prefrontal brain regions being especially affected as hypothesized by Carlsson (2001). Although there is ample evidence for an involvement of glutamatergic perturbations in OCD, it is the resulting treatment strategy that proves or disproves this concept. Therefore, it is an interesting notion that lamotrigine augmentation of selective serotonin re-uptake inhibitor (SSRI) treatment has been regarded as an effective therapeutic strategy (Bruno et al. 2012). Memantine add-on to fluvoxamine significantly improved short-term outcomes in patients with moderate to severe OCD (Ghaleiha et al. 2013; Hezel et al. 2009; Stewart et al. 2010).

Adjunctive glycine treatment (a NMDA receptor agonist) approached efficacy in OCD patients (Greenberg et al. 2009) while ketamine, a potent non-competitive NMDA receptor antagonist, has been studied in treatment-refractory OCD. Ketamine effects on OCD symptoms, in contrast to depressive symptoms, did not seem to persist or progress after the acute effects of ketamine had dissipated (Bloch et al. 2012).

The “serotonin hypothesis” of OCD has been developed in the 1980s (March et al. 1989) and was based on CSF concentrations of 5-HIAA, which were significantly increased in OCD (Insel et al. 1985). Pharmacological strategies with zimelidine, a 5-HT uptake inhibitor, reduced CSF-5-HIAA concentrations but were clinically ineffective (Insel et al. 1985). Moreover, the behavioural responses of OCD patients to m-chlorophenylpiperazine (MCPP) and tryptophan treatment had no effects on OCD symptoms (Charney et al. 1988). However, clomipramine improved symptoms worsened with metergoline, a 5-HT antagonist, in patients who had improved with clomipramine, a semiselective 5-HT uptake inhibitor (Murphy et al. 1989). MCPP, a 5-HT receptor agonist, increased anxiety, depression, and dysphoria in untreated OCD patients (Murphy et al. 1989). Regarding Cochrane analyses and meta-analyses, SSRIs are the first choice of medication for OCD in children and adults and are able to alleviate OCD symptoms significantly (Westenberg et al. 2007). Serotonin-norepinephrine re-uptake inhibitors are less effective and only third choice, but have been described as showing fewer side effects (Sansone and Sansone 2011).

All in all, SSRIs lack efficiency in ADHD while they are highly efficient in OCD. In contrast, dopaminergic and noradrenergic interventions are effective in many although not all patients with ADHD. If we summarize the involvement of various neurotransmitters in ADHD, the following rank order is envisaged: dopamine–noradrenaline–glutamate–serotonin, while for OCD, the following rank order is envisaged: serotonin–glutamate = dopamine–noradrenaline.

## Genetic aspects in OCD and ADHD

### Twin and family studies

OCD and ADHD twin and family studies indicate high familiality in both disorders (Walitza et al. 2010; Franke et al. 2012). The heritability of obsessive compulsive symptoms ranges from 0.45 to 0.65 in children and from 0.27 to 0.47 in adults (van Grootheest et al. 2005). For ADHD, the heritability was estimated to be around 70–80 % (Faraone et al. 2005). However, more recent quantitative systematic approaches, which took into account possible biases of previous twin studies (such as lack of power to detect sibling interactions and the correction used for contrast effects), concluded that genetic factors explained 60 % of the variance of ADHD (Wood and Neale 2010). Recently, it was reviewed that many twin studies vary in several phenotypic and measurement aspects, thereby strongly influencing heritability estimates, in turn indicating that one should be cautious regarding the interpretation of these results (Freitag et al. 2010). In addition, the strong evidence supporting the notion that ADHD is an extreme of a continuous trait lead Larsson et al. (2012) to investigate the genetic links in twins between the extreme and the subthreshold range of ADHD symptoms. They found a strong genetic link between the extreme and the subthreshold variation, with almost identical group heritability estimates of around 0.60 for the diagnostic (prevalence 1.78 %) and screening (prevalence 9.75 %) criteria of ADHD (Larsson et al. 2012). Moreover, Larsson et al. (2013) described high heritability of ADHD (0.88, 95 % CI 0.83–0.93) for the entire twin sample composed of over 50,000 twins, while shared environmental effects were non-significant. Similarly, in OCD, a more recent twin study (van Grootheest et al. 2008) indicated that genetic factors contributed significantly to variations in obsessive compulsive symptom liability that were dependant on age: only 27 % at the age of 12 years, but 57 % at the age of 14 years, and 54 % at the age of 16 years. There were no sex differences in heritability, while gender difference did influence prevalence (higher prevalence in girls at age 14 and 16). Only at age 12 did environmental factors shared by children from the same family contribute significantly (16 %) to individual differences in obsessive compulsive symptom scores.

Family studies showed that first-degree relatives of patients with OCD were affected by OCD considerably more frequently than relatives of healthy control subjects (Bellodi et al. 1992; Pauls et al. 1995; Nestadt et al. 2000; Hanna et al. 2005a, b; do Rosario-Campos et al. 2005). However, in a representative recent study of Steinhausen et al. (2013) no effect of age of onset on heritability was found. Family studies showed that there is a type of OCD which might be more familial and another type which is probably more de-novo and perhaps triggered more by environmental factors. Furthermore, the studies showed that early-onset OCD seems to be a more genetic and neurobiological condition. Similarly, familial studies have shown that the risk of ADHD among parents and siblings of children who had ADHD is increased starting at two-up to eightfold higher (Wilens et al. 2005). Adoption and twin studies can help to separate, although not completely, genetic from environmental factors observed in family studies (Wood and Neale 2010). Adoption studies have unequivocally found that biological relatives of children with ADHD are more likely to be hyperactive compared to adoptive relatives (Faraone and Khan 2006).

### Linkage studies

Many linkage analysis studies have been conducted in ADHD samples including child as well as adult patients with ADHD (Franke et al. 2012). Several chromosome regions have been suggested to be significantly linked to ADHD (see Table 1). A meta-analysis including 7/9 independent studies showed that the distal part of chromosome 16q is linked to ADHD (contains e.g. the CDH13 genes) (Zhou et al. 2008). This has been also shown in genome-wide association studies (GWAS, Lesch et al. 2008).

**Table 1:** Significant linkage findings from genome-wide scans and meta-analysis of ADHD and nominal significant findings in OCD

Chromosome	Chromosomal region	ADHD	OCD
1	1p21		GWL (Ross et al. 2011)
	1p36		GWL (Mathews et al. 2012)
	1q		GWL (Shugart et al. 2006) GWL (Hanna et al. 2007)
	1q25.1	GWL (Romanos et al. 2008)	
	1q25.3	GWL (Romanos et al. 2008)	
2	2p14		GWL (Mathews et al. 2012)
	2p25.1	GWL (Saviouk et al. 2011)	
	2q21.1	GWL (Rommelse et al. 2008)	
	2q35	GWL (Romanos et al. 2008)	
3	3q27–28		GWL (Shugart et al. 2006)
4	4q13.2	GWL (Arcos-Burgos et al. 2004)	
5	5p	GWL (Hebebrand et al. 2006)	
	5p13	GWL (Friedel et al. 2007)	
	5p13.1	M (Ogdie et al. 2006) GWL (Romanos et al. 2008)	
	5q13		GWL (Mathews et al. 2012)
	5q33.3	GWL (Arcos-Burgos et al. 2004)	
6	6p25		GWL (Mathews et al. 2012)
	6q		GWL (Shugart et al. 2006)
	6q22-23	GWL (Romanos et al. 2008)	
7	7p		GWL (Shugart et al. 2006)
9	9p24		GWL (Hanna et al. 2002)
	9q22	GWL (Romanos et al. 2008)	
	9q31.1–33.1	GWL (Romanos et al. 2008)	
	9q33	GWL (Romanos et al. 2008)	
10	10p13		GWL (Mathews et al. 2012)
	10p15		GWL (Hanna et al. 2007)
11	11q22	GWL (Arcos-Burgos et al. 2004)	
13	13q12.11	GWL (Rommelse et al. 2008)	
14	14q12	GWL (Romanos et al. 2008)	
15	15q		GWL (Shugart et al. 2006)
	15q11.2–13.3	GWL (Romanos et al. 2008)	
	15q14		GWL (Ross et al. 2011)
16	16p12.3–12.2	GWL (Romanos et al. 2008)	
	16p13	M (Zhou et al. 2008) GWL (Ogdie et al. 2003)	
	16q23.1–24.3	M (Zhou et al. 2008)	
	16q24		GWL (Ross et al. 2011)
17	17p11	GWL (Arcos-Burgos et al. 2004)	
	17p12		GWL (Ross et al. 2011)
18	18q11.2–12.3	GWL (Romanos et al. 2008)	
	18q21.31–21.32	GWL (Saviouk et al. 2011)	

*ADHD* attention deficit hyperactivity disorder, *GWL* genome-wide linkage study, *M* meta-analysis, *OCD* obsessive compulsive disorder

Similarly, several linkage studies (Arcos-Burgos et al. 2004; Hebebrand et al. 2006; Romanos et al. 2008) as well as one meta-analysis (Ogdie et al. 2006) indicate a significant linkage on chromosome 5 in a region containing the dopamine D1 receptor as well as the dopamine transporter genes (DAT1, SLC6A3). These candidate genes have also been found in many association studies to be significantly associated with ADHD (Nemoda et al. 2011). Therefore, we assume that these two dopaminergic genes seem to contribute to ADHD.

In comparison with ADHD, only five genome-wide linkage (GWL) studies of OCD have been published so far (see Table 1). In all five studies, no significant genome-wide evidence for linkage was detected according to standard guidelines for linkage studies (Lander and Kruglyak 1995). However, several loci displayed suggestive evidence for linkage findings. In particular, chromosome 1 seems to repeatedly show evidence for linkage with OCD (Hanna et al. 2007; Shugart et al. 2006; Ross et al. 2011; Mathews et al. 2012). Another frequent finding is from Hanna et al. (2002) who found in a second GWL scan the maximum linkage signal on chromosome 10p15. Concordantly, Mathews et al. (2012) also found a linkage signal in a nearby region (10p13). Chromosome 10 seems to show a more specific contribution to OCD since no findings were reported in ADHD (see Table 1).

### Association studies

To date, although several GWAS as well as meta-analyses for association studies in ADHD have been conducted, no single single-nucleotide polymorphisms (SNP) or polymorphism has been found to be affected in ADHD (see Table 2; Zhang et al. 2012). Furthermore, similar as in ADHD, no single SNP or polymorphism has been found to be affected in OCD, as found in the only GWAS published to date (Stewart et al. 2013b), in which no SNP reached genome-wide significance, as well as from the meta-analysis conducted by Taylor (2013). Nevertheless, it seems that dopaminergic, serotonergic, noradrenergic, synaptic, and growth factor genes are involved in ADHD, while in OCD, the serotonergic and glutamatergic genes seem to play a greater role (see Table 2).

**Table 2:** Genetic risk factors in ADHD versus OCD

	Genes	Chromosomal location	ADHD	OCD
Dopaminergic	DRD1	5q35.2	M−	n.a.
	DRD2	11q23.2	M+	M−
	DRD3	3q13.31	M−	M−
	DRD4	11p15.5	M+	M−
	DRD5	4p16.1	M+	n.a.
	TH	11p15.5	M−	n.a.
	MAOB	Xp11.3	− −	n.a.
	COMT	22q11.21	M−	M+ (men)
	DAT1 (SLC6A3)	5p15.33	M+	M−
	DDC	7p12.1	+ +	n.a.
Serotonergic	SERT (SLC6A4)	17q11.2	M+	M+
	HTR2A	13q14.2	M−	M+ (early onset)
	HTR1A	5q12.3	+	n.a.
	HTR1B	6q14.1	M+	M−
	HTR1D	1p36.12	±	+
	HTR2C	Xq23	− −	M−
	TPH2	12q21.1	M+	±
	TPH1	11p15.1	M−	–
	MAOA	Xp11.3	++	M−
Noradrenergic	NET (SLC6A2)	16q12.2	M−	–
	DBH	9q34.2	M+	n.a.
	ADRA2A	10q25.2	M−	n.a.
	ADRA2C	4p16.3	− −	n.a.
Cholinergic	CHRNA4	20q13.33	M−	n.a.
Glutamatergic/GABAergic	GRIN2A	16p13.2	− −	n.a.
	GRIK2	6q16.3-q21	n.a.	+ +
	SLC1A1	9p24	n.a.	M±
	GABRB3	15q12	n.a.	++
	SLC6A1	3p25-p24	+	n.a.
Synaptic	SNAP25	20p12.2	M+	n.a.
	CDH13	16q23.3	±	n.a.
	CTNNA2	2p12	±	n.a.
Others	BDNF	11p14.1	M−	M±
	BDKRB2	14q32	n.a.	++
	CHRM5	15q26	n.a.	++
	CHRNA1	2q31.1	n.a.	++
	UBE3A	15q11.2	n.a.	++
	TNFA	6p21.2	n.a.	++
	GLRB	4q31.3	n.a.	++
	LPHN3	4q13.1	+ +	n.a.
	ASTN2	9q33.1	±	n.a.
	OLIG2	21q22.1	n.a.	+ +
	NTRK2	9q21.33	±	+ + (female)

*M+* meta-analysis showing positive association, *M−* meta-analysis showing negative association, ± findings are contradictory, *+ +* more positive association results compared to the negative results, *+* suggestive evidence (no replication yet), *− −* more negative association results compared to the positive results, *−* single negative study, not available (n.a): PubMed search did not yield any result. For ADHD, data were extracted using the ADHDgene database (http://adhd.psych.ac.cn/index.do), Neale et al. (2010b), Shiffrin et al. (2013), Wu et al. (2012), Forero et al. (2009), Sun et al. (2013) and Gizer et al. (2009). For OCD, data were extracted from reviews of Nicolini et al. (2009), Nestadt et al. (2010), Walitza et al. (2010), Pauls (2010), Nemoda et al. (2011), Stewart et al. (2013a) and Taylor (2013)

#### Dopaminergic genes

Although SSRIs are the first-line treatment for OCD, in cases of non-response, augmentation of low-dose neuroleptics is sometimes effective (Komossa et al. 2010). This implies that there is involvement of dopamine-related genes in OCD. Nevertheless, only *COMT* showed some significant association in OCD after an extensive meta-analysis (Taylor (2013); Table 2). Clinical studies with responders and non-responders to drug treatment were stratified according to dopamine D2 Taq/A and *COMT* Val158Met genotypes (Vulink et al. 2012). There was no significant difference in genotype distribution or allele frequencies of the *COMT* or dopamine receptor D2 (*DRD2*) between responders and non-responders to citalopram with quetiapine. However, OCD patients with the Met/Met genotype (48 %) of the *COMT* polymorphisms showed a treatment response to 10-week citalopram in drug-free/drug-naive OCD patients (Vulink et al. 2012). This is in line with some clinical findings showing that lower activity of *COMT* associated with the Met allele lead to poorer executive function in OCD (Tukel et al. 2013). Recently, *COMT* mRNA was shown to be expressed significantly lower in patients with OCD compared to controls in peripheral blood samples (Wang et al. 2009). Influence of variations in the *DAT1* and *COMT* genes on neural activation during response inhibition with different activation during inhibition have been described. Inhibitory control seems therefore associated with variation of dopamine function (Congdon et al. 2009). But recent studies, genome-wide linkage scans and GWAS, are contradictory considering the “classic” dopaminergic genes like *COMT*. That resulted in no significant association with ADHD after meta-analysis (Sun et al. 2013; Zhang et al. 2012). While the *COMT*-SNP rs4680 (Val158Met) Met allele reduces *COMT* enzyme activity and is associated with impulsiveness and substance abuse in ADHD, Soeiro-De-Souza et al. (2013) compared the Met/Met genotype with the Val/Val genotype in healthy individuals. These authors demonstrated that the rs4680 Met/Met genotype was associated with higher impulsivity on the BIS-11 s-order factor Non-planning scale. Therefore, such data are in line with the suggestion that increased dopamine concentration induces impulsivity and substance abuse depending on the sensitivity of motor- and limbic circuitry systems (Soeiro-De-Souza et al. 2013). Involvement of dopamine systems in the pathology of ADHD is also evident from PET-studies with ^11^C-altropane. It could be shown that both ADHD and the 3′-UTR (*SLC6A3*) *DAT1* polymorphism had additive effects on ^11^C-altropane DAT binding (Spencer et al. 2013). Similarly, a single-photon emission computed tomography meta-analysis in healthy controls could show nominal higher levels of striatal DAT in the 9-repeat allele carriers (Costa et al. 2011). According to many association studies followed by meta-analysis, it has been shown that the 10-repeat allele of the *DAT1* is a risk allele for ADHD in childhood (Gizer et al. 2009; Table 2), while persistent ADHD in adults was associated with the 9-repeat allele (Franke et al. 2010), findings that might point to a regulatory effect of DAT in brain development. Nevertheless, such association could not be confirmed in a recent meta-analysis for OCD (Taylor 2013; Table 2), pointing to a unique effect in ADHD.

Dopaminergic receptors (D1–D5) are essential in modulating behaviour. The high risk of D2, D4, and D5 receptor polymorphisms in ADHD has been reviewed by Wu et al. (2012). Disruption of D2 signalling in the ventral striatum impairs motivation. In contrast, postsynaptic overexpression in the nucleus accumbens increases in animals the willingness to expend efforts to obtain a goal (Trifilieff et al. 2013). Furthermore, *DRD4* and tyrosine hydroxylase (*TH*) polymorphisms are associated with activity–impulsivity-related traits in dogs (Wan et al. 2013). Several studies suggest that the length of the *DRD4* repeat affects the activation of the receptor as well as the mRNA expression of the gene (Nemoda et al. 2011). Meta-analysis in ADHD revealed a significant association between the 7-repeat allele of the *DRD4* exon 3 VNTR (OR 1.33, 95 % CI 1.15–1.54) and the disorder (Gizer et al. 2009), while association of the *DRD4* 4-repeat allele and OCD was indicated by a case–control study (Camarena et al. 2007) and by a family-based study (Walitza et al. 2008), whereas an increased frequency of the *DRD4* 7-repeat allele was shown in a late-onset OCD group (Hemmings et al. 2004), and in a subgroup of OCD patients with comorbid tics (Cruz et al. 1997). Nevertheless, in a recent meta-analysis, this association to OCD could not be confirmed (Taylor 2013; Table 2). Signalling properties and regulation of DRD4 as well as the interaction of DRD4 in modulating GABAergic transmission has been reviewed by Furth et al. (2013). As the adenosine A2A receptor (ADORA2A) is linked to dopaminergic transmission, Molero et al. (2013) studied the relationship between *ADORA2A* gene polymorphisms and ADHD traits in 1,747 twins. One of the SNPs, rs35320474, showed a significant correlation to ADHD traits (Molero et al. 2013).

In conclusion, it seems that dopamine-related genes are much more associated with ADHD than with OCD (see Table 2).

#### Serotonergic genes

Selective serotonin re-uptake inhibitors block the SERT and represent the most effective pharmacological treatment of early and late OCD. Most association studies in OCD have therefore investigated serotonergic genes (see Table 2). This is in contrast to ADHD where pharmacotherapy is focused on the regulation of dopaminergic and noradrenergic dysfunction. The most frequently studied gene in OCD is *SERT* [SLC6A4], with its functional polymorphism in the upstream region termed *5*-*HTTLPR*, which involves an insertion (L-[long] allele)/deletion (S-[short] allele) polymorphism. In comparison with the S-allele, the L-allele has been reported to exert an increased transcriptional activity and an increased basal re-uptake of 5-HT in vitro (Heils et al. 1996; Lesch et al. 1996; Murphy and Lesch 2008). The L-allele is therefore referred to as the gain-of-function variant of the serotonin transporter. In contrast to anxiety disorders, it has been observed in OCD that the L-allele is associated with the disorder, in particular in early onset (Bloch et al. 2008; Taylor 2013; Walitza et al. 2005). Similarly as for OCD, the L-allele of the *SERT* was found to be significantly associated with ADHD (OR 1.17 95 % CI 1.02–1.33) (Gizer et al. 2009). This might imply the similarity in dysfunction of impulse control in both disorders, caused by dysfunctions of the serotonergic pathways. Nevertheless, some contradicting results were reported in which no significant association between *5*-*HTTLPR* and ADHD was found in a meta-analysis (Forero et al. 2009).

In line with the serotonergic hypothesis, a significant association with the rs6311 A-allele carriers of the serotonin 2A receptor (*HTR2A*) with OCD was confirmed by a meta-analysis (Taylor 2013; Table 2). However, this gene seems to be specific to OCD as no positive findings were reported for ADHD (see Table 2).

Another gene related to the serotonergic system is the brain-specific tryptophan hydroxylase-2 (*TPH2*), the rate-limiting enzyme in 5-HT synthesis in the brain, that was studied for the first time for its association in early-onset OCD (Mossner et al. 2006) and in ADHD by our groups (Walitza et al. 2005). This gene, according to a meta-analysis by Gizer et al. (2009), showed a significant association with ADHD, while it failed to associate with OCD after meta-analysis (Taylor 2013), probably because the numbers of studies with OCD are still too low.

In conclusion, it seems that the serotonin-related genes have some common gene associated with OCD and ADHD (e.g. *SERT*), although some genes seem to be unique to one disorder (e.g. *HTR2A* for OCD; *HTR1B*, *TPH2* and *MAOA* for ADHD).

#### Noradrenergic genes

Since one of the therapeutic targets of medication (such as atomoxetine) in ADHD is the norepinephrine transporter (*NET*), this gene and related genes have been studied. Yet, only the dopamine beta hydroxylase (*DBH*), the enzyme that synthesizes norepinephrine from dopamine, was found to be associated with ADHD after a meta-analysis (Gizer et al. 2009). While neither *NET* nor other noradrenergic genes were found to be associated with ADHD or with OCD (see Table 2).

#### Glutamatergic genes

The neuronal glutamate transporter (*SLC1A1*) gene on 9p24 is one of the few candidate genes for OCD that was investigated due to its localization within a linkage peak by Hanna et al. (2002). The role of this glutamate transporter gene in OCD is also supported by the observation that the anti-glutamatergic drug riluzole can be beneficial in the treatment of OCD and by the finding of elevated glutamate levels in the CSF of OCD patients (Pittenger et al. 2011). Further studies of glutamatergic neurons and SLC1A1 in particular are therefore warranted in early- and adult-onset OCD (Wu et al. 2013a, b). Identification and characterization of three alternative *SLC1A1*/EAAC1 (excitatory amino-acid transporter 1) mRNAs, P2, ex2skip, and ex11skip (Porton et al. 2013), gave evidence that all isoforms inhibit glutamate uptake from the full-length EAAC1 transporter (Porton et al. 2013). While Wang et al. (2010) did find the variant T164A in one family, these authors did not find statistical differences in genotype and allele frequencies of common SNPs in SLC1A1. All in all, however, the results on *SLC1A1* in OCD are at discrepancy. Samuels et al. (2011) genotyped 111 SNPs in or near *SLC1A1* and conducted family-based association analyses in 1,576 participants in 377 families. None of the surrounding markers were in linkage disequilibrium with rs301443 (SNP 7.5 kb downstream of the *SLC1A1* gene) nor were any associated with OCD. These authors, however, found that rs4740788 was associated with OCD in all families and in families with affected males. A three-SNP haplotype (rs4740788–rs10491734–rs10491733) was associated with OCD in the total sample (Samuels et al. 2011). *SLC1A1* rs3056 variant could be shown to be associated with increased total, left, and right thalamic volume in OCD (Arnold et al. 2009). Also, there might be a common locus for OCD and autism spectrum disorders at rs301443 residing between *SLC1A1* and JMJD2C (Lysine-specific demethylase 4C/*KDM4C*) at 9p24 (Kantojarvi et al. 2010). Non-significant trends were identified by Taylor’s meta-analysis report (2013) for the glutamate-related polymorphism rs3087879. In agreement, a much less optimistic message regarding an association between OCD and the 3′-region of *SLC1A1* is based on a recent meta-analysis (Stewart et al. 2013a). Nevertheless, the glutamatergic-related genes seem to be unique for OCD, since no significant association has to date been found in ADHD (see Table 2).

#### Synaptic genes

In regard to the synaptic genes, these have come to attention after GWAS findings in ADHD (Lasky-Su et al. 2008; Neale et al. 2010a; Neale et al. 2010b; Lesch et al. 2008). In particular the genes coding to synaptosomal-associated protein 25 (*SNAP25*), cadherin 13 H-cadherin (*CDH13*), and catenin (cadherin-associated protein) alpha-2 (*CTNNA2*) but also others (see Table 2). Indeed, *SNAP25* has been further investigated in independent studies and meta-analyses showing significant association with ADHD (Gizer et al. 2009; Galvez et al. 2013). Furthermore, in a recent study, the variant rs362990 on the *SNAP25* gene was associated with ADHD in which the risk A-allele was also associated with additive decrease in the expression of the *SNAP25* transcript in the inferior frontal gyrus of 89 unaffected adult post-mortem tissue (Hawi et al. 2013). Moreover, reduced *SNAP25* levels in developing glutamatergic synapses alter short-term plasticity, in which glutamatergic neurotransmission is enhanced (Antonucci et al. 2013). Still, the synaptic-related genes should be further investigated in more depth in order to confirm GWAS and other association studies in ADHD. In contrast, in OCD, such studies have not yet been conducted, and therefore, no conclusion in regard to their involvement in the disorder can be conveyed.

## Conclusions

OCD and ADHD are frequent psychiatric disorders which are highly comorbid with each other and with other psychiatric symptomatology. In early-onset OCD, ADHD is one of the most common comorbidities. Structural and functional imaging findings have shown abnormalities converging with a failure of CST circuit function responsible for cognitive control and performance monitoring processes in both ADHD and OCD patients.

Neuropsychological tests and corresponding brain activation studies showed, for example, deficits in response inhibition common to both disorders. According to the nature of their symptoms situated at the opposite ends of the impulsive–compulsive spectrum, either hypo- or hyperactivation of affected brain structures such as basal ganglia or the mesial frontal cortex was reported. Nevertheless, the causes of the deficits may be different. In the case of OCD, they could be caused by the overflow of intrusive thoughts, whereas in ADHD, it might be due to a lack of inhibitory control and to an impulsive response style. Abnormal inhibitory processes e.g. may therefore be responsible for both, the perseverative, compulsive symptoms characterizing patients with OCD and the disinhibited, impulsive behaviours seen in patients with ADHD. Neurochemically ADHD and OCD are varied in particular in their pathobiochemical and pathogenetic involvement of dopamine (ADHD) and serotonin (OCD). Fronto-striatal- and orbito-frontal circuitries reflect significant differences from a morphological and neurochemical point of view. The ACC and MPFC have recently received major interest especially in OCD. The molecular genetic findings correspond well to the neurochemical and imaging perspective. Linkage studies show for ADHD, regions of significant linkage on chromosome 16q, containing the *CDH13* gene and for chromosome 5 containing the *DRD1* and the *DAT1* genes, supporting the strong evidence for the involvement of the dopaminergic system in the aetiology of ADHD. In regard to OCD, no significant genome-wide evidence for linkage has been so far detected; however, there is some evidence for linkage on chromosome 1 and 10. In contrast to ADHD, especially association studies in OCD supported the contribution of serotonergic and also glutamatergic genes with the disorder. For some OCD phenotypes, augmentation of SSRIs with dopaminergic-based treatments is recommended. In the case of ADHD so far, dopaminergic- and noradrenergic-based medication remains the first choice medication. In summary, multimodal studies investigating the aetiological factors of psychiatric disorders and phenotypes have the potential to contribute to the ongoing development of more effective and specific treatment strategies not only for the different disorders but also for clinical presentations of broader phenotypes.

## Abbreviations

(f)MRI: (functional) Magnetic resonance imaging
ACC: Anterior cingulate cortex
ADHD: Attention deficit hyperactivity disorder
ADORA2A: Adenosine A2A receptor
BDNF: Brain derived neurotrophic factor
cAMP: Cyclic adenosine monophosphate
COMT: Catecholamine-O-methyltransferase
CSF: Cerebrospinal fluid
CST: Cortico-striato-thalamico-cortical
DA: Delay aversion
DAT: Dopamine transporter
DAT1 (SLC6A3): Dopamine transporter gene
DRD2: Dopamine receptor D2
DRD4: Dopamine receptor D4
EAAC1: Excitatory amino-acid transporter 1
EEG: Electroencephalography
ERN/Ne: Error-related negativity
ERP: Event-related potentials
GABA: Gamma-aminobutyric acid
GWAS: Genome-wide association studies
HTR2A: Serotonin 2A receptor
MAO-A: Monoamine-oxidase-A
MCPP: M-chlorophenylpiperazine
MEG: Magnetoencephalography
MPFC: Medial prefrontal cortex
MRS: Proton magnetic resonance spectroscopy
NAA: N-acetylaspartate
NET: Norepinephrine transporter
NGA: Nogo anteriorisation
NMDA: N-methyl D-aspartate
NST: Subthalamic nucleus
NTRK2: Neurotrophic tyrosine kinase receptor type 2
OCD: Obsessive compulsive disorder
OFC: Orbito-frontal cortex
PD: Parkinson’s disease
PET: Positron emission tomography
PFC: Prefrontal cortex
SERT (SLC6A4): Serotonin transporter
SLC1A1: Glutamate transporter gene
SMA: Supplementary motor areas
SNP: Single-nucleotide polymorphism
SPECT: Single-photon emission computed tomography
SRT: Serial reaction time
SSRI: Selective serotonin re-uptake inhibitors
SSRT: Stop signal reaction time
TH: Tyrosine hydroxylase
TPH2: Tryptophan hydroxylase-2
VNTR: Variable number tandem repeat
